# moTSart: accelerating automated transition state search with generative models in a low-data regime

**DOI:** 10.1039/d6dd00259e

**Published:** 2026-09-11

**Authors:** Leonard Galustian, Johannes Karwounopoulos, Tori Demuth, Jasper De Landsheere, Konstantin Mark, Maximilian P.-P. Kovar, Anton Zamyatin, Dennis Svatunek, Esther Heid

**Affiliations:** a Institute of Materials Chemistry, TU Wien A-1060 Vienna Austria esther.heid@tuwien.ac.at; b Institute of Applied Synthetic Chemistry, TU Wien A-1060 Vienna Austria

## Abstract

Transition states (TSs) are first-order saddle points on the potential energy surface, and thus the highest-energy structure on the (multi-step) minimum-energy reaction pathway that corresponds to a chemical reaction, determining the rate at which it proceeds. Locating TSs and thus elucidating reaction mechanisms is a computationally demanding and expertise-driven task. Recent automation strategies leverage heuristic rules or deep learning models to directly arrive at TS geometries from textual SMILES representations. However, standalone TS predictors lack robustness, whereas heuristic-based approaches lack scalability, limiting their applicability to reliable high-throughput reaction discovery under distribution shifts. Here, we present a generative machine learning framework embedded within a fully automated TS search pipeline and demonstrate accelerated high-throughput screening of bioorthogonal click reactions as a representative case study. The automated framework enables scalable exploration of large reaction spaces and progressively learns, reducing the number of required quantum mechanical evaluations as additional reactions are processed. In our case study, generative refinement decreased the required TS optimization cycles by nearly 40% after training on only 544 reactions. By coupling generative modeling with physics-based validation in a scalable workflow, our work establishes a framework for data-driven reaction mechanism exploration and advances the development of autonomous computational discovery of chemical reactivity.

## Abstract 2

We introduce moTSart, a framework for generative AI accelerated, automated transition state search.

## Graphical abstract

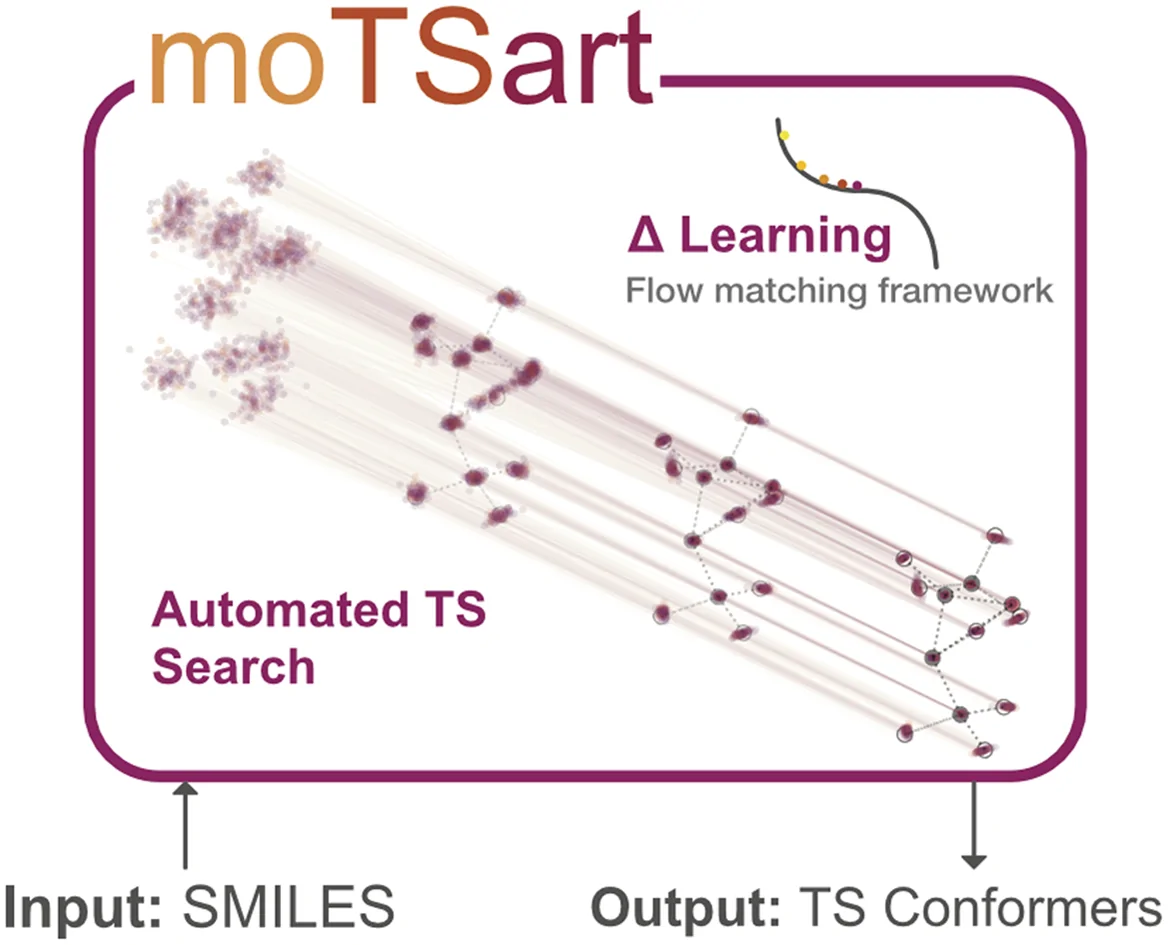

## 1 Introduction

Accelerating automated reaction profile calculations can speed up the discovery of a vast number of molecules, ranging from life-saving drugs to environmentally friendly organocatalysts.^1^ An important prerequisite for calculating reaction barrier heights is knowledge of the transition state (TS), which is an index-1 saddle point on the potential energy surface, connecting two local minima, namely specific reactant and product conformers of an elementary-step chemical reaction.^2^ Elucidating mechanisms of novel reactions often requires years of human intuition and domain expertise to guide the discovery process. In this sense, mechanistic discovery is frequently regarded as much an art as it is a science. This duality is reflected in the project name, motsart.

Approaches for *ab initio* TS search can be broadly categorized into single-ended^3^ and double-ended methods.^4,5^ In this work, we focus on double-ended methods since we assume knowledge of both reactant and product minima and their atom-mapping, provided as atom-mapped SMILES strings, which drastically reduces the search space.^6^ These algorithms locate the minimum energy path (MEP), where the highest energy image along the path approximates the TS. After locating this approximate TS, it must be saddle point optimized to a structure containing exactly one negative imaginary frequency, ideally followed by an intrinsic reaction coordinate (IRC) validation to confirm that the TS connects the intended reactants and products.^2^

Machine learning (ML)-based approaches to TS search can directly generate the TS structure using generative methods, given only a SMILES string as input,^7–10^ and optionally a starting guess.^11,12^ Recent work has also started tackling distribution shifts.^13,14^ In general, a drawback of current generative models in molecular modeling, including protein folding, is their tendency to predict geometrically accurate but physically implausible structures caused, *e.g.*, by steric clashes.^9,15–17^

Machine learning interatomic potentials (MLIPs) can also be used to estimate reaction paths and saddle point-optimize the resulting TS guesses^18,19^ and they have recently become highly competitive to DFT-based approaches for automated TS search.^20–22^ Generative approaches are complementary: a physics-based workflow produces paired input and output geometries that can be used to train an amortized, stage-specific generative model. This model can bypass the expensive search or optimization process by directly predicting the outputs of those calculations in milliseconds. As computational backends improve, the rate-limiting stage may change, and the corresponding source and target distributions of the generative model can be redefined accordingly.

Partial automation of the TS search process has been pursued by several research groups,^1,23–26^ commercial software,^27^ and applied in practical problem settings.^28–30^

Another potentially costly stage in high-throughput workflows is IRC validation. Its purpose is to validate if a discovered TS is connected to the intended reactants and products. Recent work has introduced graphRC, a graph-based method that bypasses expensive IRC calculations by analyzing internal coordinate changes for a multitude of reaction types.^31^ The approach requires only a single Hessian calculation, thereby validating TS connectivity in seconds rather than hours, given a converged TS optimization. Rapidly sampling TS conformers while keeping the reaction core frozen is another key component, for which an efficient open-source method, racerTS, was recently developed.^32^

The aim of this work is to establish a framework that integrates generative ML methods to accelerate TS discovery for bimolecular organic chemical reactions and to provide a systematic, scalable procedure that eliminates trial-and-error human intervention. Furthermore, we propose methods to incorporate the latest research on high-throughput TS search into the automated framework. We validate our method by demonstrating its ability to accelerate high-throughput screening of bioorthogonal click reactions as a representative case study.

## 2 Methods

Our framework consists of three components:

1. complexfinder: searching for reactant complexes that lead to low-energy TSs.

2. pathguesser: finding minimum-energy paths connecting these reactant complexes to their respective products.

3. validator: performing saddle point optimizations and IRC validations to arrive at the TS connecting these reactants and products.

**Fig. 1:**
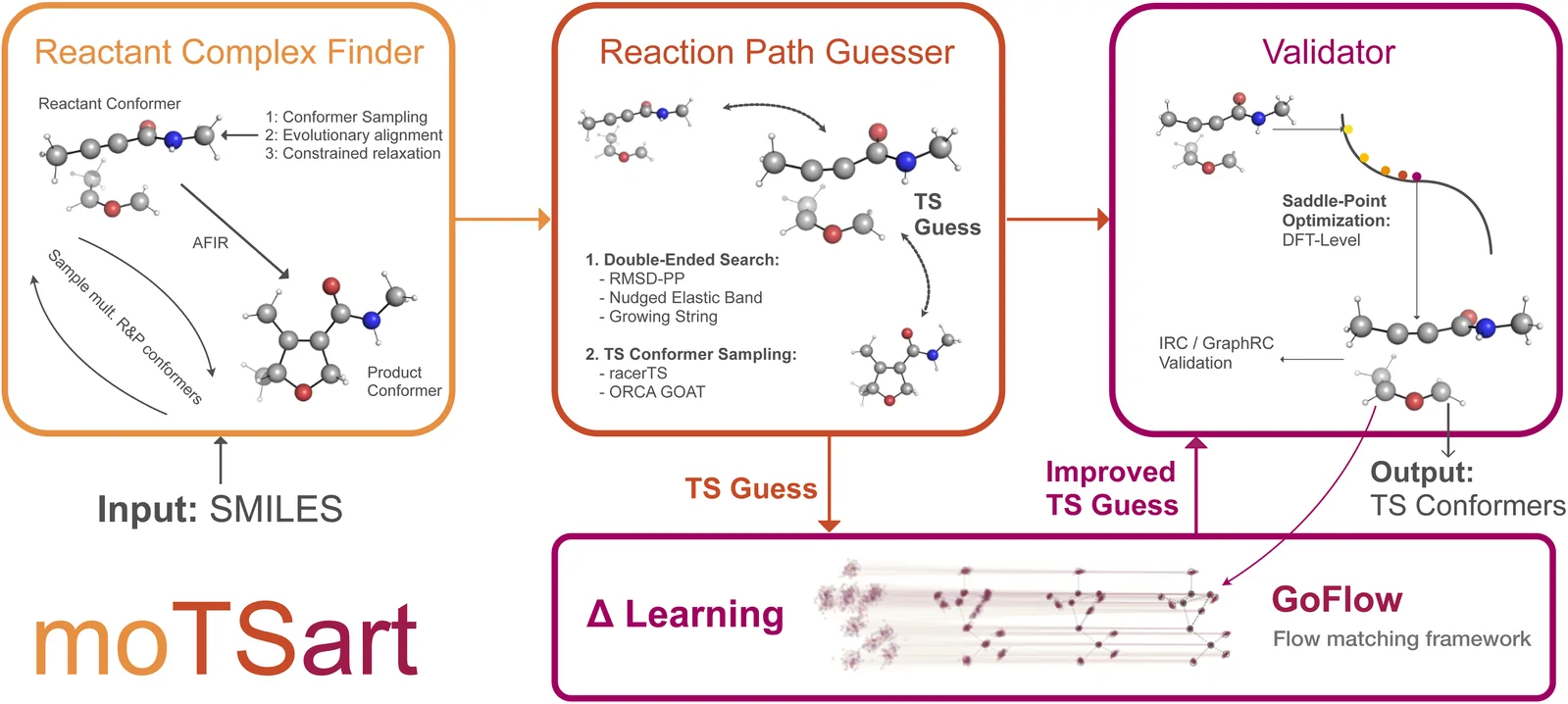
The four components of the motsart framework. Reactant Complex Finder: samples reactant (R) complexes using an evolutionary algorithm and obtains their respective product (P) conformers with AFIR. Reaction Path Guesser: uses xTB double-ended reaction path search (based on R&P conformers) to obtain TS guesses. Validator: saddle point optimizes the TS guesses with DFT or an MLIP and performs endpoint validation (IRC or graphRC). Delta learning: in the cycloaddition experiments studied here, the model takes TS guesses from the pathguesser as its source distribution and saddle point-optimized geometries from the validator as its target distribution. This specific instantiation is termed tsoptnet.

Each of those steps can be accelerated with a generative model at different stages of reaction processing. In this work, we focus on the saddle-point-optimization component. Currently, this is performed in an online learning setting; however, an active learning approach could also be employed to increase sampling efficiency. The entire procedure is visualized in Fig. 1.

### 2.1 Reactant complex search

Aligning reacting molecules, both spatially and conformationally, so that an energetically relevant reaction occurs and finding the corresponding aligned product, is a challenging endeavor.^1,25,33^ Well-aligned reactant and product pairs are required for subsequent double-ended reaction path search algorithms, such as the double-ended growing string method or the nudged elastic band method, to converge towards meaningful MEPs. As the size of the reactants increases, this can become a computationally expensive search problem. Moreover, the lowest-energy conformer of a reactant or intermediate is not necessarily associated with the lowest reaction barrier; higher-energy conformers can, in some cases, lead to lower activation barriers. Consequently, obtaining representative reaction energy profiles requires calculating and evaluating TSs for multiple reactive conformers.^2^ This systematic exploration of reactive conformational space is a central component of the motsart framework.

We split the reactant complex search into three stages and term this module complexfinder. First, intramolecular conformers of the separated reactants are generated. Second, an evolutionary algorithm (EA) searches over the relative rigid-body rotations and translations of the reactant fragments to obtain sterically feasible reactive complexes with appropriate forming-bond distances. Third, the best complexes are relaxed using constrained semiempirical geometry optimizations and subsequently passed to the AFIR-based product/path-guessing stage. Detailed pseudocode is provided in the SI. Reaction network exploration frameworks like YARP^25^ approach reactant complex search by generating unconstrained, independently sampled reactant and product conformer pools and subsequently performing force-field-driven joint optimization to transform their structures into matching topologies.

#### 2.1.1 Evolutionary search

We use an evolutionary search strategy to rapidly generate a conformationally diverse set of reactant molecules where the reacting atoms are approximately spaced apart by the sum of their summed Van-der-Waals (VdW) radii, multiplied by a scaling factor, with no steric clashes occurring. Optionally, evolutionary pressure can be applied to the angle alignments of the reactive core atoms to favor geometries close to the product structure. The initial conformers are generated with the ETKDGv3 algorithm of RDKit.^34^ The EA then treats each conformer as a candidate reactant complex and mutates only the relative placement of the molecular fragments by applying random rigid-body rotations and translations to each reactant molecule.

Each candidate is scored by a penalty function containing three terms: a forming-bond distance penalty, a steric-clash penalty between atoms belonging to different reactant fragments, and an optional product-similarity penalty based on angles around the reacting atoms. The forming-bond penalty drives newly forming atom pairs toward a target distance proportional to the sum of their VdW radii, whereas the clash term removes complexes with unphysical intermolecular overlaps. The product-similarity term compares local reactive-core angles in the reactant complex to those in a relaxed product reference geometry, thereby biasing the search toward orientations that are geometrically compatible with the target product if desired by the user. After scoring, the lowest-penalty candidates are retained as elites, while the remaining population is generated by tournament selection followed by mutation. The mutation intensity is gradually annealed over generations to transition from global exploration to local refinement.

Tournament selection is used to filter, replicate, and subsequently mutate the next generation. We found tournament selection ideal, since the population maintains diversity even after hundreds of evolutionary generations, as shown in an analysis using the cycloaddition dataset in the SI. As a useful chemical heuristic, we selectively filter for reactant conformers whose dihedral angles are close to those of the respective product. This heuristic is particularly useful for the cycloaddition reactions, which we aim to study in this work. As an illustrative example, take the Diels–Alder reaction, where the Diene must be in the higher-energy s-cis conformation to react with the Dienophile.^35^

#### 2.1.2 Constrained relaxation

We perform a subsequent relaxation for the evolutionarily fittest reactive alignments. This is particularly necessary for low-barrier reactions, where unrelaxed reactant complexes can have energies higher than those of their subsequently obtained TS. This step is performed using a constrained relaxation with GFN2-xTB,^36^ where bond-forming atoms are constrained to remain at a specific distance. This distance is the sum of the atoms VdW radii, multiplied by an application-specific factor.

#### 2.1.3 AFIR product alignment

To obtain the accordingly aligned products, corresponding to the obtained reactant complexes we employ a similar strategy to TS-Tools for performing the AFIR search.^24,37^ The force constant for pushing the bond-forming atoms closer in the constrained relaxation process is instead chosen using a more efficient binary search, which scales logarithmically. This process naturally produces a reaction path with a corresponding TS guess structure. However, we found that double-ended methods such as the meta-dynamics-based RMSD-push-pull method by Grimme^38^ resulted in a larger number of successful saddle point optimizations for the corresponding TS guesses. The obtained reactant and product structures are thus passed to a path-guessing module, which performs fast double-ended TS search using a relatively low level of theory such as GFN2-xTB. The pathguesser module is described next.

### 2.2 Reaction path calculation

Given aligned reactant and product complex geometries, a double-ended reaction path calculator can be used to calculate an approximation of the MEP connecting those two local minima on the potential energy surface.^4^ This MEP must pass through a saddle point, or multiple saddle points in case of a multi-step reaction. Popular and established methods are the nudged elastic band method^5^ and the (double-ended) growing string method.^39^ While highly accurate when used with a high level of theory DFT backend, they are computationally too expensive for screening hundreds of reactions.

Using faster but less accurate semi-empirical methods to obtain a reaction path guess and subsequently saddle-point-optimizing the respective TS guess with a more expensive method has been proposed in previous works for high-throughput applications.^26^ The authors showed particularly reliable results, compared to DFT, for low-barrier reactions of less than 30 kcal mol^−1^, using the RMSD-PP method^38^ for obtaining reaction path guesses.

We adopt this strategy to generate reaction path guesses, since the dataset of study, curated by Stuyver *et al.*,^40^ consists of [3 + 2] cycloaddition reactions with mostly low activation barriers. In early experiments, we found this method to yield more reliable results than using the AFIR path that is obtained in the AFIR product alignment step described in Section 2.1.

Importantly, our implementation of motsart is modular, allowing researchers to seamlessly integrate their preferred reaction path calculators into the pipeline *via* convenient abstract base classes. By default, users can choose between the AFIR, RMSD-pp, CI-NEB, and ML-FSM path guessers, with CI-NEB and ML-FSM using the eSEN-OMol25-sm-conserving MLIP.^41,42^

#### 2.2.1 TS conformer sampling

The second step in the pathguesser module concerns sampling low-energy TS conformers from the TS conformer obtained by a reaction path calculator. We use the recently released racerTS method by Schmid *et al.*^32^ to sample TS conformers. The algorithm returns a set of diverse TS conformers, which we then constrain-relax at the GFN2-xTB level of theory, keeping the reaction core frozen. Subsequently we sort the obtained conformers by their energies and pass the lowest energy conformers to the validator module. This last constrained relaxation and energy sorting step reduced the number of subsequent saddle point optimization cycles required in early experiments. It costs an additional several seconds, depending on the reaction, and can be turned off optionally.

### 2.3 Validation

The validator module is responsible for performing saddle point optimizations on the TS guesses obtained from the pathguesser and validating them *via* IRC calculations. We implemented four distinct backends for these tasks: (1) a semi-empirical GFN2-xTB variant using the ORCA^43^ interface, (2) a DFT variant also based on ORCA, (3) an MLIP variant using the ORCA external methods interface, and (4) a GPU-accelerated implementation based on GPU4PySCF^44,45^ using the software suite pysisyphus for optimization algorithms.^46^ The latter (4) is particularly efficient for Hessian calculations, making it highly effective for these validation steps. However, for the cycloaddition experiments reported here, we employed the ORCA DFT module because CPU resources were readily available at the time of the study, and DFT level energies are often preferred over faster, approximate methods.

#### 2.3.1 Saddle-point optimization

Saddle-point optimizations were performed at the B3LYP-D4/def2-TZVP level of theory using the RIJCOSX approximation with the def2/J auxiliary basis set. The optimization utilizes the partitioned rational function optimization (P-RFO) algorithm. Stationary points were confirmed as first-order saddle points by frequency analysis, ensuring the presence of exactly one imaginary frequency corresponding to the reaction coordinate.

#### 2.3.2 IRC validation with graphRC

The validator module has built-in support of the recently released graphRC library.^31^ Their package requires a QM frequency output file as input, parsing the vibrational normal modes to analyze connectivity changes along the saddle point. They validated the approach for pericyclic reactions, demonstrating 100% accuracy in detecting primary bond changes for diverse ring-closing and Diels–Alder systems.

We use these detected bond changes as a connectivity test: a TS is accepted when every bond change expected from the atom-mapped reaction is recovered by graphRC. This is a necessary condition for the saddle point to connect the intended reactants and products. By default, we do not additionally reject a TS for extra bond changes that graphRC reports but the reaction template does not list, since the imaginary-mode analysis can occasionally flag transient spectator contacts in asynchronous or loosely coupled motions. This default proved sufficient for the reactions studied here, since in every case examined, an incorrect saddle point failed due to dissociation of the reacting fragments during TS optimization, which removes an expected bond and is therefore detected as a missing change. We never observed a TS that passed while forming an unintended bond. Enabling the stricter setting, requiring the detected and expected bond-change sets to be identical, introduced only false rejections, as the flagged extra contacts were spectator interactions.

For the pericyclic reactions examined here, this heuristic validation provided a faster alternative to full IRC calculations without an observed loss of accuracy. If the stricter set-equality criterion or full IRC calculations are required—for instance, for reaction classes where unintended bond formation is a plausible failure channel—motsart provides options to enable them.

### 2.4 Generative modeling

Each geometry-producing module of motsart can, in principle, be augmented by a generative model trained on paired input and output geometries. In the cycloaddition workflow studied here, we focus on the validator because DFT saddle-point optimization was the most expensive geometry-refinement stage under the computational setup employed for that case study. We train tsoptnet, a goflowv2-based model (Section 2.4.2), to approximate the corresponding input–output transformation and to provide improved initial geometries for subsequent physics-based optimization and validation.

To facilitate future research on accelerating the other modules, we also release an auxiliary parameter-efficient multi-head version of goflow^9^ which leverages a shared backbone network and three small task-specific networks to jointly generate reactant complexes (for reactants and products) and a TS guess, using a cross-attention module to exchange information between the task-specific networks. It is readily integrated into the motsart pipeline. Note, however, that the reported experimental results do not use this model.

We constructed the initial dataset for tsoptnet by performing DFT saddle point optimizations on TS guesses generated by the pathguesser module. The generative model is thus trained to perform a delta-learning task:^17,47^ transforming rough, GFN2-xTB-level TS guess structures into refined, DFT-level saddle-point-optimized geometries. The final dataset comprises the first 681 reactions from the [3 + 2] cycloaddition dataset^40^ with the lowest atom counts, filtered to include only those successfully validated by graphRC. This data was divided into an 80/10/10 train/validation/test split (544/68/69 reactions). For each reaction, we computed 2–3 distinct TS conformers, yielding a total of 1510 conformers with final split sizes of 1214/146/150. Crucially, the splits were stratified by reaction to ensure that no conformers from the same reaction appeared across the training, validation, or test sets.

We chose to extend the goflow^9^ method for generative modeling, since it is highly flexible for reaction modeling, allowing us to convert any start distribution (at *t* = 0), such as Gaussian noise, or any geometry guess distribution into a target distribution (at *t* = 1) of interest. The underlying E(3)-equivariant gotennet architecture that predicts the velocity vector *v*_*θ*_(*x*_*t*_|*x*_0_) makes it significantly more data-efficient and faster than previous architectures across diverse data regimes.^48^

Before describing the goflowv2 architectural enhancements, we briefly review the basics of flow matching in the context of tsoptnet.

#### 2.4.1 Flow matching

Let *p*_0_(*x*) be the source distribution and *p*_1_(*x*) be the target data distribution. While standard generative frameworks define *p*_0_ as a standard normal distribution 
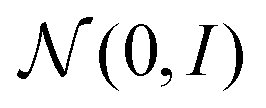
, for the tsoptnet delta-learning task, *p*_0_ represents the distribution of rough TS guesses. For tsoptnet, *p*_1_ corresponds to DFT-optimized saddle points. The flow-matching framework constructs a probability trajectory *p*_*t*_(*x*) over *t* ∈ [0,1], connecting the initial source distribution *p*_0_ to the target *p*_1_. A time-dependent vector field *v*_*t*_(*x*) governs the transition along this path. The continuous evolution of a state *x*_*t*_ ∼ *p*_*t*_ is defined by the underlying ordinary differential equation (ODE):^49^
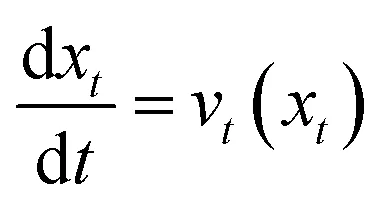


Flow matching aims to train a parameterized neural network *v*_*θ*_(*x*,*t*), in this work, the E(3)-equivariant gotennet architecture, to approximate the vector field *v*_*t*_(*x*).

Because the exact marginal probability path *p*_*t*_(*x*) remains intractable, modeling *v*_*t*_(*x*) directly is practically not possible. Conditional Flow Matching (CFM) bypasses this limitation by constructing a surrogate target vector field derived from exact conditional trajectories.^49^ By coupling specific source and target samples, *x*_0_ ∼ *p*_0_ and *x*_1_ ∼ *p*_1_, it creates a trajectory *via* linear interpolation:*x*_*t*_ = (1 − *t*)*x*_0_ + *tx*_1_

The time derivative of this conditional path yields a constant vector field:
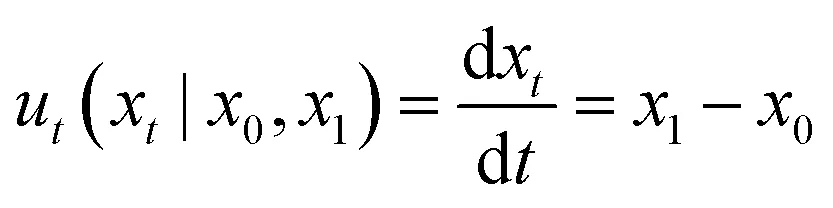


Since this target field evaluates to (*x*_1_ − *x*_0_), it is invariant with respect to both time *t* and the intermediate state *x*_*t*_. From a delta-learning perspective, this target field represents the geometric displacement induced by the selected workflow transformation. In the tsoptnet instance studied here, it is the displacement from a pathguesser TS guess to the corresponding DFT-optimized saddle-point geometry.

Optimization of the network *v*_*θ*_(*x*,*t*) relies on computing the expected mean squared error between the model predictions and the true conditional displacement, averaged across both time *t* and the paired distributions (*x*_0_,*x*_1_):
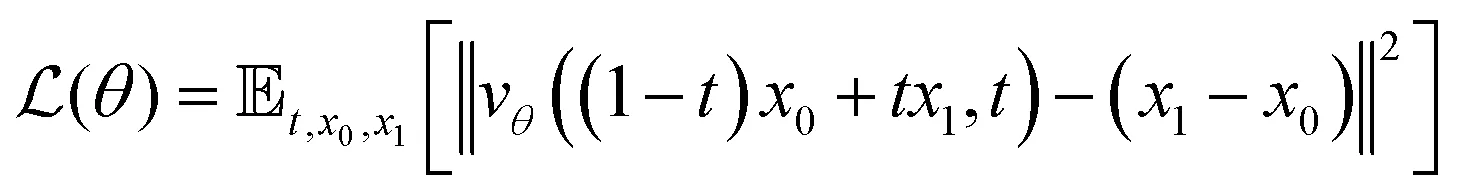
with 
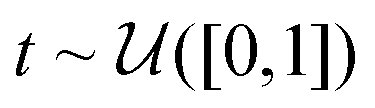
, *x*_0_ ∼ *p*_0_, and *x*_1_ ∼ *p*_1_. Minimizing this objective results in *v*_*θ*_(*x*,*t*) approximating the marginal vector field *v*_*t*_(*x*).^50^

During inference, generating samples representing the target distribution *p*_1_(*x*) requires evaluating the learned ODE. Starting from a rough initial guess *x*_0_ ∼ *p*_0_(*x*), we propagate the system forward in time from *t* = 0 to *t* = 1:
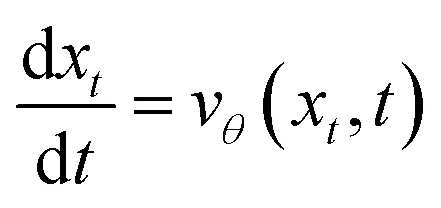


Standard numerical ODE solvers can be used for this simulation. In this work, we employ the Euler method. The resulting state *x*_1_ at *t* = 1 approximates the target distribution *p*_1_(*x*), corresponding to a refined DFT-level saddle point geometry.

#### 2.4.2 GoFlowV2

In the standard flow-matching formulation described above, the network must regress the target vector field *u*_*t*_ = *x*_1_ − *x*_0_ given only the intermediate state *x*_*t*_ and time *t*. This poses an inverse problem, as the network must implicitly reconstruct the reference geometry *x*_0_ to determine the correct update direction. This suggests that for non-Gaussian *p*_0_, conditioning on the de-noised prior could facilitate learning, which we found to be the case. In our setting, *p*_0_ is either the sampled RDKit geometry during pre-training or the TS guess to be saddle-point-optimized during fine-tuning.

The priorconditioning module computes the atom-wise displacement vectors *d*_*i*_(*t*) = *x*_*i*_(*t*) − *x*_*i*_(0) for each atom *i*. To maintain the E(3)-equivariance of the network, we decompose this displacement into its invariant norm *D*_*i*_ = ‖*d*_*i*_(*t*)‖ and its equivariant unit direction *û*_*i*_ = *d*_*i*_(*t*)/(*D*_*i*_ + *ε*). These geometric quantities are then injected directly into the network's node features before the first interaction layer.

Let 
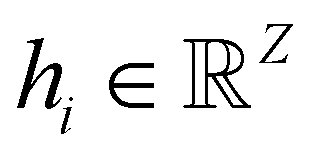
 be the scalar node features and 
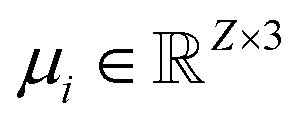
 be the vector features. The conditioning is applied additively:

1 *h*^′^_*i*_ = *h*_*i*_ + MLP_s_(*D*_*i*_)

2 µ^′^_*i*_ = *µ*_*i*_ + (*û*_*i*_ ⊗ MLP_v_(*D*_*i*_))

where MLP_s_ and MLP_v_ are multi-layer perceptrons that project the displacement magnitude into the feature dimension *Z*, and ⊗ denotes broadcasting the scalar weights across the vector direction.

#### 2.4.3 Training stages

##### 2.4.3.1 Pre-training

We pre-train the TS prediction model on the Reaction-QM (RQM) dataset,^51^ specifically the B3LYP-D3/TZVP level of theory optimized subset, which contains almost 197 000 reactions. Pre-training results are shown in the SI.

##### 2.4.3.2 Fine-tuning stage 1: domain adaptation

To facilitate domain adaptation to a new dataset of interest, we fine-tune the model on 1000 reactions, totaling 2500 TS guesses from the pathguesser module. These data points are inexpensive to generate compared to the higher-level DFT data obtained from the validation module.

Previous work has shown that features such as bond lengths are not learned accurately when trained on small TS datasets.^9,14^ When adapting to new TS prediction datasets involving novel chemistry, Darouich *et al.*^14^ showed that training on high-energy reactant conformers, termed “pseudo-TSs” in their work, improves domain adaptation. With motsart, it is now possible to rapidly generate those TS guesses. They are significantly closer to the true TS than high-energy reactant conformers. This stage, in addition to the pre-training, is particularly important in a low-data setting, where high-quality DFT data is scarce.

##### 2.4.3.3 Fine-tuning stage 2: TS optimization

This stage first requires obtaining DFT-level saddle points by running the validator module on a specified number of reactions. The generative model is then fine-tuned to optimize the TS guess towards the saddle point. Neural network weights are initialized from fine-tuning stage 1. The TS guesses form the starting distribution of the model at *t* = 0, and the corresponding saddle points form the data distribution at *t* = 1. We refer to the resulting model as tsoptnet.

Table 1 summarizes the source and target distributions used in each training and inference stage. In the TS-prediction stages, the source geometry is constructed as the atom-wise midpoint between the RDKit reactant and product conformers, optionally perturbed by Gaussian noise during training. In the tsoptnet delta-learning stage, the source distribution is instead the pathguesser TS-guess distribution, and the target distribution is the corresponding DFT saddle-point geometry.

**Table 1:** Source and target geometries used in the flow-matching stages of the moTSart framework. *R*_RDKit_ and *P*_RDKit_ denote RDKit-generated reactant and product conformers, respectively. *x*_PG_ denotes the TS guess obtained from the pathguesser module, and *x*^‡^_DFT_ denotes the corresponding DFT-level saddle point-optimized TS. Gaussian noise is added only during training

Stage	Task	Source geometry (*p*_0_) at (*t* = 0)	Target geometry (*p*_1_) at (*t* = 1)
Pre-training	TS prediction on RQM	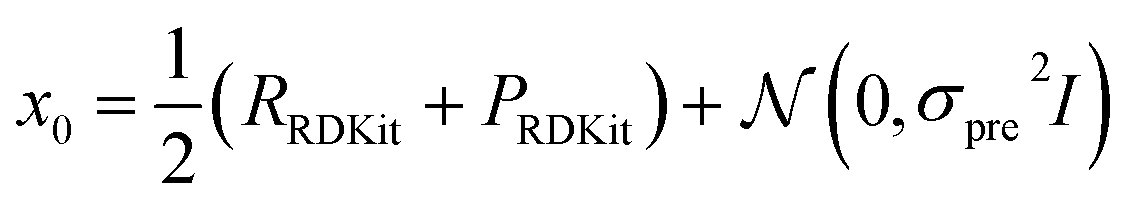	*x*_1_ = *x*^‡^_RQM_ RQM TS
Fine-tuning 1	Domain adaptation to the target reaction class	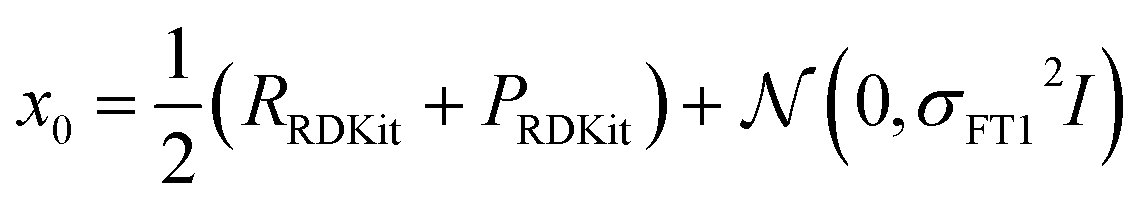	*x*_1_ = *x*_PG_ TS guess from pathguesser
Fine-tuning 2	TsOptNet delta learning	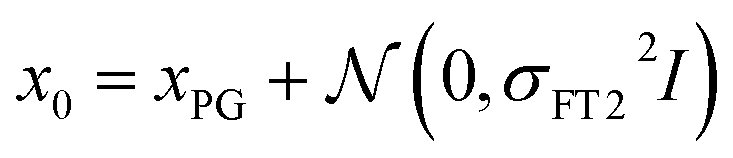	*x*_1_ = *x*^‡^_DFT_ DFT-level saddle point-optimized TS
Inference: RQM TS prediction	—		*x*_1_ = *x*^‡^_RQM_ RQM TS
Inference: TS refinement	TsOptNet refinement before DFT optimization	*x*_0_ = *x*_PG_	*x*_1_ = *x*^‡^_DFT_ DFT-level refined TS guess

## 3 Results and discussion

### 3.1 Generative modeling

#### 3.1.1 Geometric evaluation

**Fig. 2:**
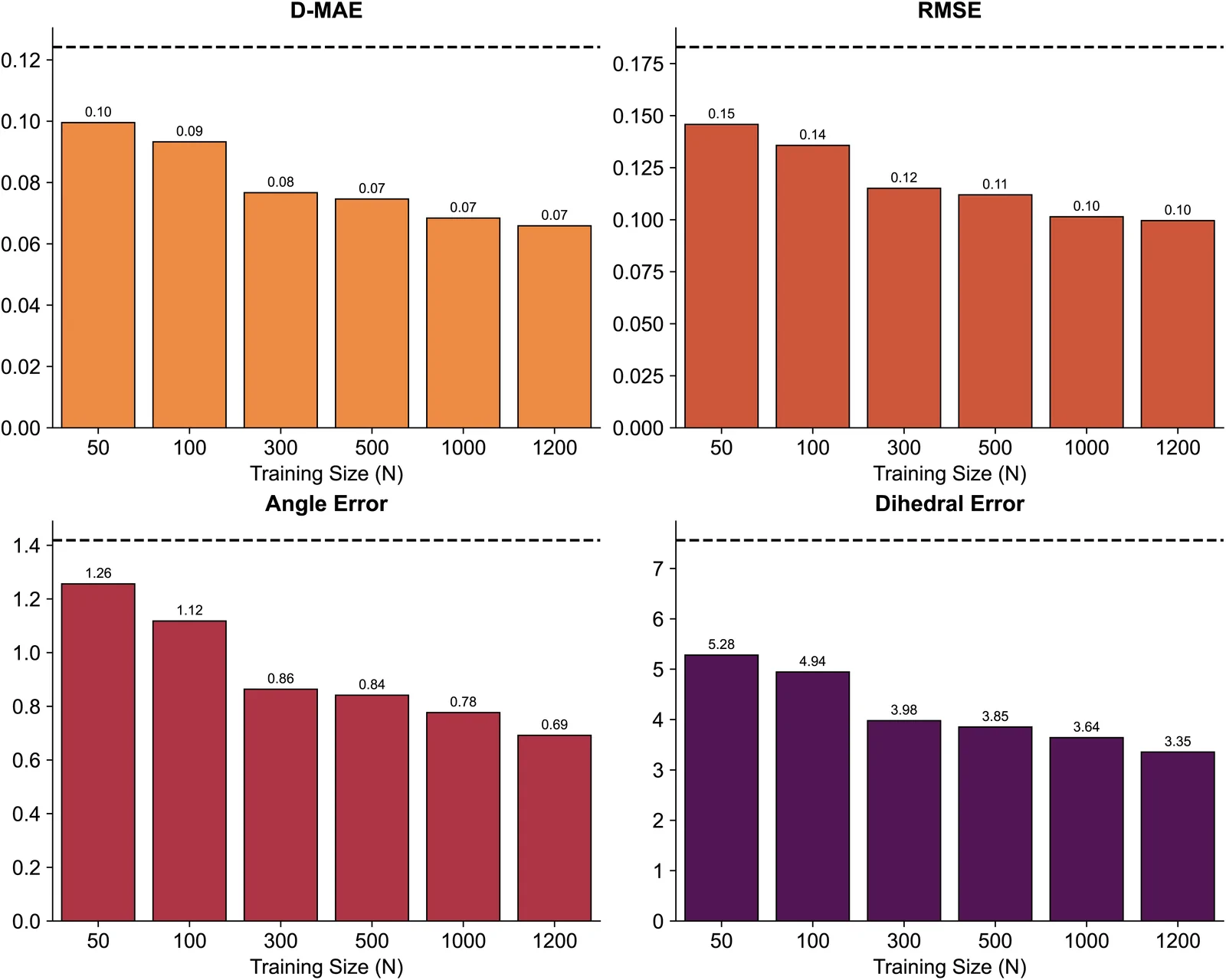
Mean absolute error of interatomic distances (D-MAE) [Å], RMSE [Å], angle error [°], and dihedral error [°], averaged across the test set. DFT-level saddle-point-optimized structures serve as ground truth. Predictions are performed with tsoptnet using training set sizes ranging from 50 to 1200 TS conformers (N). The dashed black line shows the results of the pathguesser module (GFN2-xTB level TS guesses and sampling performed using racerTS with frozen reaction core). Those geometries were optimized with tsoptnet to yield the given results.

Geometry prediction metrics for tsoptnet are shown in Fig. 2. The figure summarizes six fine-tuning runs, in which the training set size was increased from 50 to 1200 TS conformers. This corresponds to 25 to 544 unique reactions, as 2–3 TS conformers were calculated per reaction. We find that additionally examining angle and dihedral errors provides a more comprehensive assessment of geometry prediction performance. All evaluated metrics–mean absolute error of interatomic distances (D-MAE), RMSE, angle error, and dihedral error–decrease with increasing data set size. Notably, significant performance improvements are achieved using only 50 data points, compared to the TS guess from the pathguesser that the model optimizes, and that serves as the baseline. Comprehensive pre-training results on the RQM dataset are presented in the SI.

Adding low-*σ* Gaussian noise to the *p*_0_ TS guess distribution for tsoptnet improved model performance. However, this required prior conditioning, since valuable information from the TS guess would otherwise be lost during the noise-addition process. We hypothesize that adding noise helped in part due to error accumulation during Euler integration and an imperfectly learned velocity network *v*_*θ*_ that indirectly adds noise during integration. This is mitigated when the model also observes a noisy probability path *p*_*t*_(*x*) during training. A similar phenomenon has been observed for Diffusion models.^52^ We leave more rigorous experimental and theoretical analysis of this phenomenon to future work. A shorter ablation study on this phenomenon is presented in the SI.

#### 3.1.2 Force evaluation

Generative models for TS structure prediction, as well as in other domains such as protein folding,^53^ have been shown to produce energetically implausible geometries while still achieving seemingly low geometric errors.^9,15,16^ To investigate this issue, we analyze the distribution of mean and maximum atomic forces for each TS using GFN2-xTB in order to search for potentially large force components arising from physically unrealistic geometries. This provides a more comprehensive assessment of potential failure modes beyond purely geometric metrics.

**Fig. 3:**
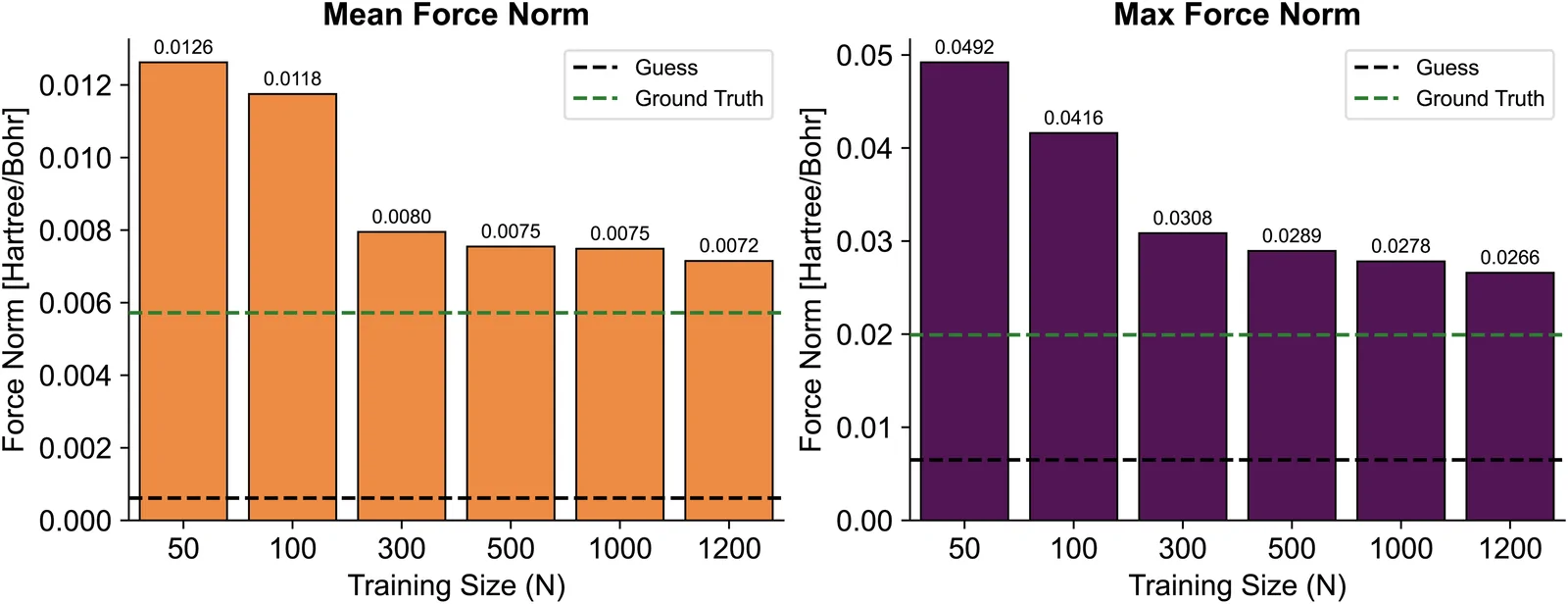
Mean and maximum forces across all atoms of the molecules, averaged across the test set, at the GFN2-xTB level of theory. Predictions are performed with tsoptnet, with varying training set size, from 50 to 1200 TS conformers (*N*) (corresponding to approx. 25 to 544 unique reactions). The dashed black line shows the forces of the pathguesser module. Those geometries were optimized with tsoptnet to yield the given results. Importantly, note that the forces are calculated with GFN2-xTB. The TS guesses by the pathguesser are obtained using the same level of theory. It is thus easier for those structures to reach low forces on the exact potential energy surface for which they were optimized. This can be observed in the ground-truth DFT saddle points having a higher force norm than the xTB-level guess.

The average forces across all TSs in the test set for tsoptnet are shown in Fig. 3 for each training set size. We do not observe large forces due to physically unrealistic geometries. As the training set size increases, the predicted structures continuously exhibit smaller forces that approach those of the ground-truth DFT TSs. Note that the DFT reference structures exhibit higher forces under GFN2-xTB evaluation than the TS guess structures, as the latter were optimized using GFN2-xTB – the same method employed for the force evaluation.

In this experiment, we observed no large GFN2-xTB force outliers in the delta-refined structures. This is consistent with the expectation that initializing generative models from distributions that are geometrically close to the target structure and energetically plausible improves prediction quality.

#### 3.1.3 QM evaluation

We compared DFT saddle-point optimization starting from the same 150 pathguesser TS guesses without refinement, after tsoptnet refinement, and after *in vacuo* saddle-point optimization with eSEN-OMol25-sm-conserving using Sella. The MLIP baseline used no reaction-class-specific fine-tuning. All resulting geometries underwent the same B3LYP-D4/def2-TZVP/SMD(water) P-RFO optimization and validation procedure, irrespective of whether the preceding MLIP optimization yielded a valid first-order saddle point.

As shown in Table 2, tsoptnet, trained on 544 reactions, reduced the mean number of subsequent DFT optimization cycles from 19.21 to 11.46, compared with 14.92 after MLIP refinement. Successful DFT optimization and validation were obtained for 147/150 tsoptnet-refined guesses and 133/150 MLIP-refined guesses. These results support the benefit of the refinement learned at the target level of theory within the class of cycloaddition reactions studied here. MLIP refinement settings are provided in the SI.

**Table 2:** DFT saddle-point optimization at B3LYP-D4/def2-TZVP/SMD(water), starting from the same 150 test-set TS guesses (69 reactions), with no refinement, tsoptnet refinement, or MLIP refinement. Cycle statistics are calculated over successful optimizations. MAD denotes mean absolute deviation. Success requires a converged first-order saddle point passing the graphRC connectivity check

Method	Cycles mean ± MAD	Median	Success
pathguesser	19.21 ± 6.52	16	150/150^a^
tsoptnet	11.46 ± 3.64	12	147/150
MLIP TS Opt	14.92 ± 4.30	13	133/150^b^

a The test set contains only guesses successfully optimized and validated by the unrefined baseline.

b Includes seven coupled-perturbed SCF failures. Excluding these, MLIP-refined guesses succeeded in 133/143 cases.

#### 3.1.4 Improved and worsened predictions

To better understand the model's behavior, we examine four cases in which tsoptnet-refined TSs required either fewer or more optimization cycles than pathguesser. We observe that tsoptnet performs significant corrections, particularly in the bonding regions, either increasing or decreasing partial bond lengths at the TS. Visualizations and additional information are shown in the SI.

#### 3.1.5 Extensions

The motsart package provides researchers with the tools needed to readily use the framework in an end-to-end online-learning setting. Pre-trained models on RQM are provided. As previously shown, the computational overhead of DFT calculations is greatly reduced after the initial fine-tuning calculations are performed.

For efficient training, we recommend performing at least a batch of calculations before fine-tuning tsoptnet for tens of epochs. Exact numbers for the tasks presented are provided in the SI.

A further extension that could improve data-efficiency and accelerate the screening process is the incorporation of active learning. An active learning framework would use an uncertainty metric to selectively prioritize the most informative or least certain samples for evaluation.

### 3.2 Cycloaddition case studies

To show the practical utility of motsart in discovering TS geometries for concerted reactions, we first applied it to the click reactions of a 1,2,4,5-tetrazine with (a) dibenzocyclooctyne (DIBO) and (b) cyclopropane-fused dibenzocyclooctyne (DMBO) in an inverse electron-demand Diels–Alder cycloaddition. These reactions are of particular interest due to their bioorthogonality and their distinctive reactivity, enabling rapid and selective transformations under physiological conditions without interfering with native biological processes.

**Fig. 4:**
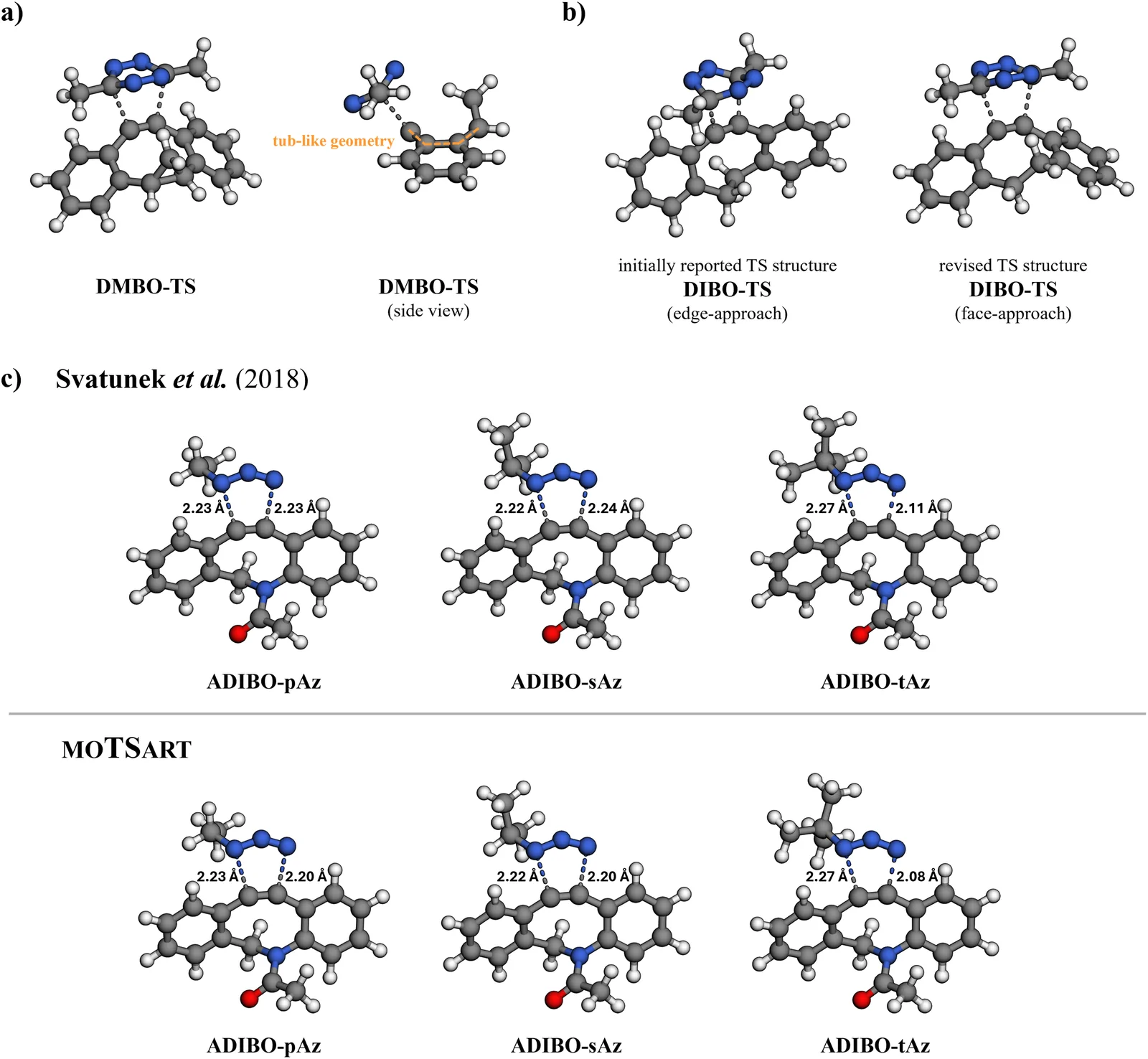
Optimized transition state (TS) geometries of (a) the reaction between 3,6-dimethyl-1,2,4,5-tetrazine and cyclopropane-fused dibenzocyclooctyne (DMBO-TS) shown from two sides, with the tub-like geometry highlighted in orange, and (b) the reaction between 3,6-dimethyl-1,2,4,5-tetrazine and dibenzocyclooctyne (DIBO-TS), both the initially reported geometry (edge-approach) as well as the revised geometry (face-approach) shown. (c) TS geometries for the reaction of 3,6-dimethyl-1,2,4,5-tetrazine with ethyl azide (pAz), isopropyl azide (sAz), and *tert*-butyl azide (tAz). The upper set of geometries is taken from Svatunek *et al.*, while the lower set was obtained using motsart. Forming bond lengths are reported in Å.

Dibenzoannulated cyclooctynes were believed to typically be non-reactive towards 3,6-disubstituted tetrazines. Houk *et al.* identified the steric demand of the benzo groups as the origin of this low reactivity.^54^ Lang and coworkers showed that the derivative DMBO exhibits unusually high reactivity toward tetrazines.^55^ This was later investigated by the same group computationally, where they revealed that the reaction proceeds *via* a tub-like TS geometry enabled by the cyclopropane fusion (Fig. 4a).^55^ This conformation significantly reduces the distortion energy of the dibenzocyclooctyne framework, thereby lowering the overall activation barrier compared to non-fused systems.

In the same study, the TS geometry of the reaction between DIBO and substituted tetrazines was revised. Earlier work had considered only a single TS conformer in which the dienophile approaches along the edge of the π-system.^54^ Lang and coworkers found that there is a second conformer in which the dienophile approaches from the side, also termed the “face” approach (Fig. 4b), demonstrating that commonly applied search strategies may overlook relevant TS structures.

motsart was able to both find the DMBO-TS with the same lowest energy tub-like conformer, as well as automatically identify both edge- and face-oriented TS conformers for the DIBO-TS within a single search (Fig. 4). In addition, the face-approach was correctly identified as the lowest energy pathway. This represents a key strength of the methodology, as it avoids bias and yields the lowest-energy TS directly from minimal input.

A second case study is based on a previous study investigating steric demand in strain-promoted alkyne–azide cycloadditions.^56^ In this study, the reactivity of primary, secondary, and tertiary azides toward cyclooctynes bicyclo[6.1.0]non-4-yne (BCN) and azadibenzocyclooctyne (ADIBO) was analyzed. It was shown that, while all azides react similarly with less sterically demanding cyclooctynes, the reactivity of tertiary azides toward bulky systems such as ADIBO decreases by several orders of magnitude due to increased steric repulsion in the TS.

Applying motsart to this system, we automatically located the TSs for the reactions of ADIBO with primary, secondary, and tertiary azides. The resulting geometries are in excellent agreement with those reported in the literature (shown in Fig. 4c), reproducing both the synchronous TSs for primary and secondary azides as well as the more asynchronous geometry observed for tertiary azides. Together, these systems span two challenges in TS discovery. In both cases, motsart successfully identified the relevant TS conformations, reproducing both unexpected structural motifs as well as mechanistic distinctions within a unified, automated framework.

### 3.3 Pipeline analysis with *S*_N_2 and *E*2 reactions

In this section, we further analyze pipeline stage-level robustness for *S*_N_2 and *E*2 reactions from the QMrxn20 dataset.^57^ We use the eSEN-OMol25-sm-conserving MLIP for path search, saddle point optimization, and IRC validation. For saddle point optimization and IRC validation, we benchmarked the Sella optimizer against ORCA ExtOpt with the integrated MLIP. Calculations are performed *in vacuo*, resembling the QMrxn20 dataset. This analysis also aims to demonstrate the MLIP capabilities of motsart.

The original QMrxn20 study identified automated TS search as a bottleneck in this reaction space, noting a low TS-search success rate, although no attempt-normalized success rate was reported. Their dataset construction relied on a mechanism-specific high-throughput protocol, including *E*2- and *S*_N_2-specific normal-mode and IRC checks, as well as reuse of validated TSs from related reactions as starting geometries for missing cases.^57^ We randomly sampled 57 × 10^2^ and 71 *S*_N_2 reactions from the dataset and searched for their TSs. The ML-FSM (machine-learning freezing string method) method^58^ was employed for path search, and saddle point optimization was performed using ORCA.^43^

A reaction counts as *solved* if the calculations yield at least one IRC-validated TS connecting the reactant and product. We initially sampled three reactant complexes per reaction for path search and saddle point optimization. The success rate can be increased by sampling more reactant complexes, albeit at a higher computational cost.

The results are shown in Table 3. When sampling and optimizing three TS guesses, *S*_N_2 reactions are solved at 84.5% and *E*2 at 54.4%. For the *E*2 reactions, the dominant failure mode is a failed path search rather than a failed saddle point optimization. For 16 of 57 × 10^2^ reactions, the path search failed, while 7 reached a converged saddle point after which the IRC failed to connect the reactant and product correctly. We conducted further analysis of the successful and failed *E*2 calculations and found that the majority of successfully optimized and validated TSs are, as expected, in the anti-periplanar conformation. The plotted distribution is shown in the SI. For 8 reactions in which both syn and anti TSs were found, the anti-periplanar TSs were, on average, 12 kcal mol^−1^ lower in energy. This result shows a potential disadvantage of unbiased sampling for this reaction type. Computational effort was devoted to energetically high, and therefore statistically unlikely, pathways, at the expense of identifying every possible TS. Future work might incorporate generic reactant complex geometry templates for specific reaction classes that favor appropriately aligned structures during the evolutionary optimization stage.

**Table 3:** Pipeline outcome by mechanism using ORCA ExtOpt. Each reaction is assigned to the first stage it fails. Path search (no TS guess produced), TS opt. (TS guess was obtained, but no TS converged), or IRC (TS opt. converged but no IRC connected reactant and product). Solved reactions yield ≥1 IRC-validated TS. Three TS guesses are sampled and optimized, except for the *E*2-6 rows, which sample six TS guesses. The *E*2-6-No-F^−^ row removes reactions with F^−^ as LG or base, and the *S*_N_2-No-F^−^ row removes reactions with F^−^ as LG

	Failed at stage				
Rxn	Path search	TS opt	IRC	Solved	Success
*E*2	16	3	7	31	54.4%
*E*2-6	5	2	11	39	68.4%
*E*2-6-No-F^−^	2	0	1	20	87.0%
*S*_N_2	8	1	2	60	84.5%
*S*_N_2-No-F^−^	4	0	2	50	89.3%

When sampling and optimizing six TS guesses, *E*2 reactions are solved at 68.4%. This shows that reaction coverage can be improved at the cost of additional sampling rounds. Notably, the success rate increases drastically when removing *E*2 reactions involving F^−^ as the leaving group or base from the dataset. This is to be expected chemically, since fluoride only qualifies as a weak Brønsted-base, leading to high-energy transition states as well as undesirable reaction energetics. Similarly, the success rate increases when *S*_N_2 reactions in which F^−^ is the leaving group are removed from the dataset. Note that F^−^ as nucleophile is acceptable for *S*_N_2 reactions *in vacuo*.

We also found that graphRC yielded inaccurate results for the *E*2 reactions studied, prompting us to perform full IRC validations for all *S*_N_2 and *E*2 reactions.

Table 4 shows the timings across the three pipeline stages for both the Sella and ORCA ExtOpt optimizers. The timing distribution strongly depends on the computational backend and its software integration. With ORCA ExtOpt, the validator dominates the wall-clock cost, whereas with Sella, the bottleneck shifts to the complexfinder. Sella is two orders of magnitude faster in this experimental setup, in part due to the overhead of the ExtOpt interface.

**Table 4:** Distribution of wall-clock time across the pipeline stages for the two saddle-point optimization engines, over the same 128 reactions and the same ML-FSM TS guesses. Median times are per reaction, counting the TS guesses that converged to a TS. A reaction processes up to three reactant complexes. Shares are of the total pipeline wall-clock, including failed attempts

	ORCA ExtOpt		Sella (ASE)	
Stage	Median (s)	Share	Median (s)	Share
Complex finder	963	27.7%	963	91.0%
Path search	42	1.2%	42	4.0%
TS opt.	1344	40.6%	23.3	2.1%
IRC	1395	30.5%	38.3	2.9%

For complexfinder, the source distribution would be RDKit-generated reactant conformers with randomized relative fragment placements. Joint targets would be the constrained-relaxed, energy-selected reactant complexes and their corresponding AFIR-generated product geometries. The released multi-head goflowv2 jointly generates reactant, product, and TS geometries, in place of these procedures. This integration provides the basis to accelerate the complex finder, although its accuracy and wall-clock speedup were not benchmarked here. Furthermore, exact amounts of data required and hyperparameter settings depend on the systems studied and are subject to experimentation.

### 3.4 Limitations and future work

In its current form, motsart is primarily validated on elementary-step bimolecular organic reactions. We demonstrated that the delta-learning framework can accelerate an iterative geometry-refinement operation by learning from its paired input and output structures. The newly developed goflowv2 architecture allows analogous source and target distributions to be defined for other geometry-producing pipeline stages. Extending the framework to study multi-step reactions, intramolecular, radical, or organometallic reactions are possible next steps towards a truly general pipeline.

Furthermore, the rise of next-generation semi-empirical methods^59^ and MLIPs^41^ can significantly accelerate data generation and screening. Benchmarking and integrating the multi-head goflowv2 architecture, or similar methods, to also accelerate those steps is a further promising direction. Also note that in a regime of sufficient in-distribution training data, a direct SMILES-to-TS generation model can be utilized. In such a setting, the complex finder and path guesser modules are not required to be run. A study analyzing how much and which data is sufficient for such models to work effectively across different reaction types and system sizes is also a promising direction for future work.

The current work focuses on accurate TS structure optimization *via* generative modeling, which forms the basis for computing reaction barriers. To obtain Gibbs free-energy barriers (Δ*G*^‡^), the minimum-energy conformers of the reactant must first be found and optimized at the chosen electronic-structure level. Harmonic vibrational frequency calculations are then performed to compute thermochemical corrections within the Rigid-Rotor-Harmonic-Oscillator approximation. Gibbs free energies are obtained by combining electronic energies with zero-point and thermal contributions derived from statistical thermodynamics, and reaction barriers are calculated as the free energy difference between the TS and the corresponding reactant minimum.^2^ Incorporating these calculation procedures into motsart, ideally including methods to accelerate them using learning algorithms, would enable automated analysis of reaction kinetics.

## 4 Conclusion

We introduced motsart, a modular framework for automated transition state discovery that integrates heuristic search strategies, quantum chemical validation, and generative machine learning. The workflow systematically identifies reactant complexes for elementary-step bimolecular organic reactions, approximates reaction paths, and validates transition states while minimizing trial-and-error human intervention and computational cost through efficient conformer sampling and fast connectivity checks.

To accelerate the pipeline, we introduced goflowv2 with priorconditioning, a data-efficient conditional flow-matching architecture for learning transformations between paired molecular-geometry distributions. This approach enables the framework to progressively accelerate calculations as additional reactions are processed. In the application studied here, the resulting model, termed tsoptnet, mapped TS guesses toward DFT-optimized saddle-point geometries and reduced the mean number of subsequent DFT optimization cycles by approximately 40%.

Overall, motsart demonstrates how combining traditional reaction-search tools with generative refinement and physics-based validation enables scalable exploration of chemical reaction space.

## Author contributions

LG: conceptualization, formal analysis, investigation, methodology, project administration, software, validation, visualization, writing – original draft. JK: investigation, software, validation. TD: visualization, formal analysis, writing – review and editing. JD: investigation. KM: investigation. MK: investigation, writing – review and editing. AZ: investigation. DS: visualization, formal analysis, writing – review and editing. EH: conceptualization, funding acquisition, project administration, supervision, writing – review and editing.

## Conflicts of interest

There are no conflicts to declare.

## Supplementary Material

DD-005-D6DD00259E-s001

## Data Availability

The motsart code is freely available at https://github.com/heid-lab/motsart. All data as well as the archived version of motsart to reproduce results is available on Zenodo 10.5281/zenodo.19554844. Supplementary information (SI): complex finder algorithm, population diversity analysis, evaluation metrics, simulation/training details, description of multi-head GoFlow, additional results. See DOI: https://doi.org/10.1039/d6dd00259e.
