# Supplementary material for: moTSart: accelerating automated transition state search with generative models in a low-data regime
Source: Digit Discov. 2026 Sep 11;5(10):4499–511. doi: 10.1039/d6dd00259e (PMC13592236; doi:10.1039/d6dd00259e)
Supplement: Supplementary file 1 [file DD-005-D6DD00259E-s001.pdf]

Supplementary Information (SI) for Digital Discovery.
This journal is © The Royal Society of Chemistry 2026

Supplementary Information:
moTSart: Accelerating Automated Transition State Search with Generative Models in a Low-Data Regime

Leonard Galustian,[1] Johannes Karwounopoulos,[1] Tori Demuth,[2] Jasper De Landsheere,[1] Konstantin Mark,[1] Maximilian P.-P. Kovar,[1] Anton Zamyatin,[1] Dennis Svatunek,[2] and Esther Heid[1, a)]

[1)] *Institute of Materials Chemistry, TU Wien, A-1060 Vienna, Austria*

[2)] *Institute of Applied Synthetic Chemistry, TU Wien, A-1060 Vienna, Austria*

[a)] Electronic mail: esther.heid@tuwien.ac.at

I. COMPLEX FINDER

Algorithm 1: ComplexFinder: reactant complex search

Input : Reactant/product graphs R, P with atom maps; bond-forming pairs B_f; VdW radii $\{r_i^{\text{vdw}}\}$; hyperparameters (population size, generations G, EA rounds N_{EA}, VdW coefficient c_{vdw}, product-similarity weight λ)

Output: Reactant complexes, TS guesses, and reaction paths

$\mathbf{x}^P \leftarrow$ relaxed reference conformer of product P

$\mathcal{C} \leftarrow \emptyset$

for $n \leftarrow 1$ **to** N_{EA} **do**

  `// Step 1: conformer generation + evolutionary search`

  Generate reactant conformers;

  Initialize population by random rigid-body rotation/translation of the kept conformers

  for $g \leftarrow 1$ **to** G **do**

    foreach *individual* $\mathbf{y}$ **do**

      $f_{\text{bond}} \leftarrow \sum_{(i,j)\in B_f} \left(\|\mathbf{y}_i - \mathbf{y}_j\| - c_{\text{vdw}}(r_i^{\text{vdw}} + r_j^{\text{vdw}})\right)^2$

      $f_{\text{clash}} \leftarrow \#\{\text{intermolecular pairs closer than } \frac{1}{2}(r_i^{\text{vdw}} + r_j^{\text{vdw}})\}$

      $f_{\text{prod}} \leftarrow \lambda \cdot (\text{reacting-atom angle deviation from } \mathbf{x}^P)$

      $f \leftarrow f_{\text{bond}} + f_{\text{clash}} + f_{\text{prod}}$

    end

    Keep the lowest-f elites; sample the rest by tournament selection

    Mutate non-elites by rigid-body rotation/translation with annealed step size

  end

  `// Step 2: constrained relaxation`

  Relax the best arrangements under bond-forming distance constraints; add the lowest-energy valid complex to $\mathcal{C}$

end

A. Algorithm

Here, we provide more detailed pseudocode in Algorithm 1, describing the complex finder module. To keep the code concise, we omitted the AFIR path search description. Note that it is technically part of the module as well.

To assemble each new generation, the non-elite slots are filled by tournament selection: for every slot, k individuals are drawn uniformly at random from the current population, and the one with the lowest penalty f is selected for the next generation. Repeating this independently for all slots biases sampling towards fitter arrangements while still allowing weaker individuals to occasionally win, which preserves population diversity over many generations. Combined with elitism, which carries a user-specified amount of lowest-penalty individuals over unchanged, this ensures that the best reactant arrangements are never lost between generations.

B. Population Diversity Analysis

To analyze tournament selection population diversity, we ran the evolutionary search for 300 generations on 16 reactions randomly sampled from the cycloaddition dataset, and tracked the population diversity at every generation. As a baseline, we repeated each run using greedy selection, in which parents are drawn only from the top 10% of the population. Both schemes were initialized identically, so any difference is attributable solely to selection. Diversity is quantified by the number of distinct structural clusters of the population. At each generation, we draw a random subsample of 256 members, align their heavy atoms onto the lowest-penalty member by the Kabsch algorithm, and compute all pairwise heavy-atom RMSDs. The members are then grouped by greedy leader clustering: iterating through the subsample, each member that lies within 1 Å RMSD of an existing cluster representative is assigned to it, and every member that does not is assigned a new cluster. The resulting cluster count measures how many structurally distinct arrangements the population contains, ranging from 256 down to 1.

As summarized in Table S1 and shown in Figure S1, both schemes start from an identical, diverse population and achieve the same optimization quality, yet tournament selection retains more structural diversity, with a median of 32 versus 18 distinct clusters after 300

Table S1. Population diversity and optimization quality of the evolutionary search under tournament and greedy selection over 300 generations. Values are medians over 16 reactions. The initial generation is identical for both schemes.

	Initial	Tournament	Greedy
Distinct clusters	250	**32**	18
Clusters (% of initial)	100%	**13%**	7%
Best penalty (a.u.)	13.56	2.91	2.97

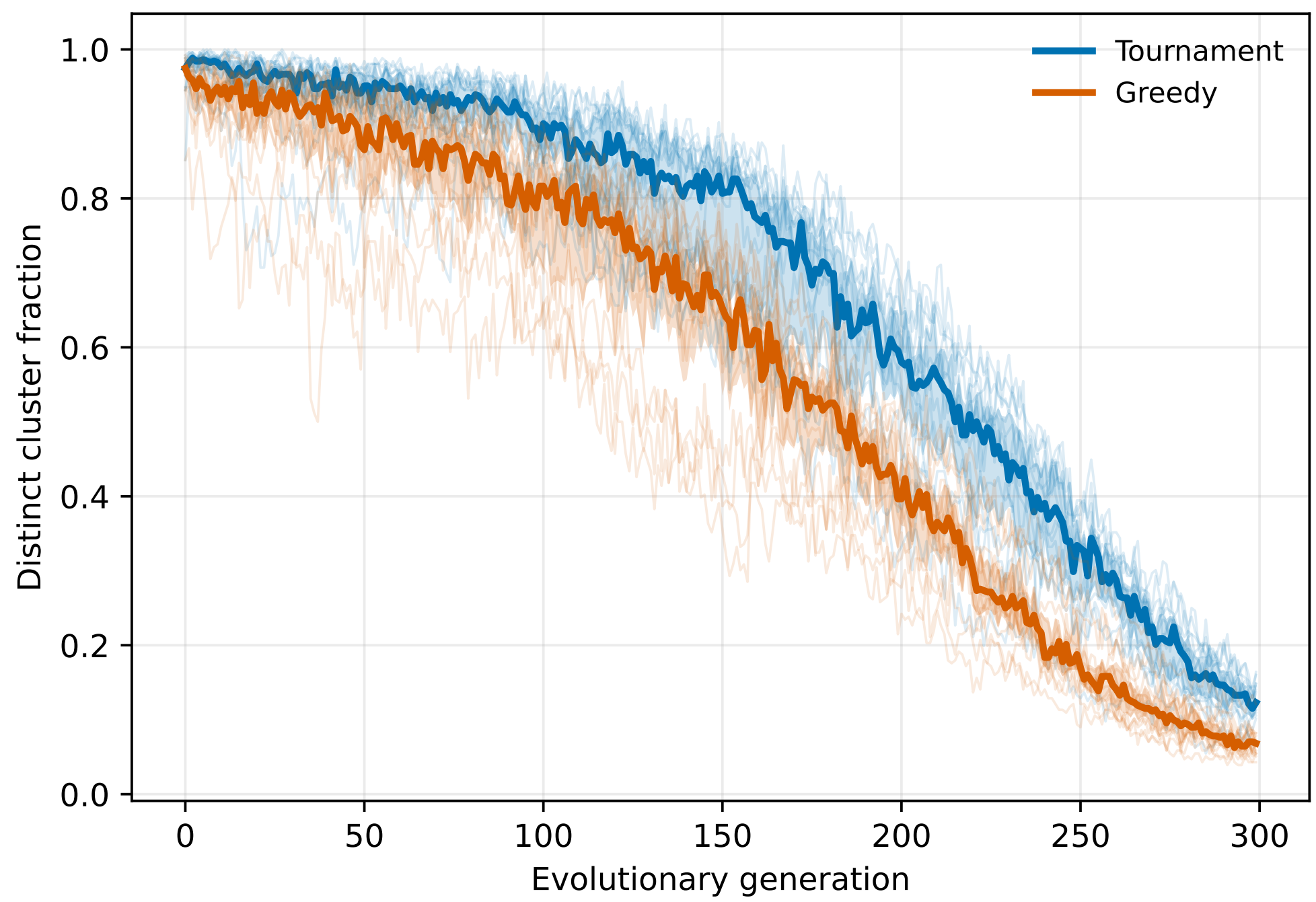

Figure S1. Population diversity of the evolutionary search as a function of generation, for tournament and greedy selection. Diversity is the distinct cluster fraction. Solid lines are the median, and faint lines are the individual reactions.

generations. In all 16 reactions, tournament selection found more distinct clusters than greedy selection did. Note that the overall decline in diversity over generations is expected, since the mutation step sizes are annealed towards the end of the search to 20% of their initial value, which contracts the population around the optimum irrespective of the selection scheme.

II. EVALUATION METRICS

Below, we define the D-MAE, RMSE, bond angle, and dihedral error metrics used to evaluate the methods.

The **D-MAE** is defined as

$$\text{D-MAE}(\mathbf{R}^{\text{pred}}, \mathbf{R}^{\text{gt}}) = \frac{2}{N(N-1)} \sum_{i<j} \left| d_{ij}^{\text{pred}} - d_{ij}^{\text{gt}} \right|, \tag{1}$$

where N is the number of atoms, and d_{ij} the Euclidean distance between atoms i and j.

The **RMSE** is defined as

$$\text{RMSE}(\mathbf{R}^{\text{pred}}, \mathbf{R}^{\text{gt}}) = \sqrt{\frac{1}{3N} \sum_{i=1}^{N} \left[(r_{x,i}^{\text{pred}} - r_{x,i}^{\text{gt}})^2 + (r_{y,i}^{\text{pred}} - r_{y,i}^{\text{gt}})^2 + (r_{z,i}^{\text{pred}} - r_{z,i}^{\text{gt}})^2 \right]} \tag{2}$$

where $\mathbf{r}^i \in \mathbb{R}^3$ are the Cartesian coordinates of atom i.

The **angle error** is defined as

$$\text{AngleError}(\mathbf{R}^{\text{pred}}, \mathbf{R}^{\text{gt}}) = \frac{1}{M} \sum_{(i,j,k)\in\mathcal{A}} \left| \theta_{ijk}^{\text{pred}} - \theta_{ijk}^{\text{gt}} \right| \cdot \frac{180}{\pi}, \tag{3}$$

where

$$\theta_{ijk} = \arccos\left(\frac{(\mathbf{r}_i - \mathbf{r}_j) \cdot (\mathbf{r}_k - \mathbf{r}_j)}{\|\mathbf{r}_i - \mathbf{r}_j\|_2 \, \|\mathbf{r}_k - \mathbf{r}_j\|_2} \right), \tag{4}$$

and M is the number of angles θ_{ijk}, defined by the set of all triplets of atoms $(i, j, k) \in \mathcal{A}$ in the structure.

The **dihedral error** is defined as

$$\text{DihedralError}(\mathbf{R}^{\text{pred}}, \mathbf{R}^{\text{gt}}) = \frac{1}{M} \sum_{(i,j,k,l)\in\mathcal{D}} \min\left(|\delta_{ijkl}|, 2\pi - |\delta_{ijkl}|\right) \cdot \frac{180}{\pi}, \tag{5}$$

where $\delta_{ijkl} = \phi_{ijkl}^{\text{pred}} - \phi_{ijkl}^{\text{gt}}$, M is the number of dihedral angles defined by the set of quadruplets $(i, j, k, l) \in \mathcal{D}$, and the angle $\phi_{ijkl} \in (-\pi, \pi]$ is computed as

$$\phi_{ijkl} = \text{atan2}\left((\mathbf{n}_1 \times \hat{\mathbf{b}}_1) \cdot \mathbf{n}_2,\ \mathbf{n}_1 \cdot \mathbf{n}_2 \right). \tag{6}$$

Here, $\mathbf{b}_0 = \mathbf{r}_j - \mathbf{r}_i$, $\mathbf{b}_1 = \mathbf{r}_k - \mathbf{r}_j$, and $\mathbf{b}_2 = \mathbf{r}_l - \mathbf{r}_k$ are the bond vectors. The unit vector of the central bond is $\hat{\mathbf{b}}_1 = \mathbf{b}_1/\|\mathbf{b}_1\|$, and the unit normal vectors to the planes are $\mathbf{n}_1 = \frac{\mathbf{b}_0 \times \mathbf{b}_1}{\|\mathbf{b}_0 \times \mathbf{b}_1\|}$ and $\mathbf{n}_2 = \frac{\mathbf{b}_1 \times \mathbf{b}_2}{\|\mathbf{b}_1 \times \mathbf{b}_2\|}$.

III. SIMULATION AND TRAINING DETAILS

Hyperparameters and additional training details are shown in Table S5. The provided table shows the default parameters for joint RQM dataset pre-training. For fine-tuning TsOptNet, we used a prior noise of $\sigma = 0.1$. Changing prior noise for fine-tuning between $\sigma \in [0.1, 0.5]$ barely affected performance. All training and inference runs were performed on a single NVIDIA A100 GPU. The best pre-training results on RQM were achieved after 140 epochs of multi-head training and 75 epochs of TS-only training. The best fine-tuning results for TsOptNet were achieved after 59 epochs.

ORCA version 6.1.1 was used for all QM calculations.

MLIP saddle-point refinement. The 150 test-set TS guesses were refined in vacuo using Sella 2.5.0 and the eSEN-OMol25-sm-conserving potential. The total charge was determined from the reaction SMILES, and the spin multiplicity was set to 1. Sella used `order=1`, internal coordinates, and initial-Hessian refinement, with a maximum-force convergence threshold of 0.01 eV,Å^{-1} and a limit of 300 steps. If internal-coordinate initialization failed, Cartesian coordinates were used with otherwise identical settings. The remaining Sella parameters were left at their defaults.

IV. MULTI-HEAD GOFLOW

We extended GoFlow by replacing a single velocity network with a parameter-efficient *multi-head* architecture. Concretely, for each task/head $h \in \{\mathrm{R}, \mathrm{TS}, \mathrm{P}\}$ we evolve a head-specific coordinate tensor $x_t^{(h)} \in \mathbb{R}^{N\times 3}$ along an optimal-transport (OT) conditional path and predict a corresponding velocity field $v_\theta^{(h)}(x_t^{(h)}, t)$ using (i) a shared E(3)-equivariant backbone and (ii) small head-specific refinement blocks. The head-specific blocks are optionally coupled via cross-attention, allowing the TS head to attend to reactant/product features. To the node features, we also add a learned embedding that specifies the input type $T \in \{\mathrm{R}, \mathrm{TS}, \mathrm{P}\}$. The flow-matching objective remains the OT conditional flow-matching

loss.

For the multi-head architecture, which predicts reactant and product complexes and a TS guess, the initial distribution p_0 represents randomly sampled RDKit conformers, plus Gaussian noise $\mathcal{N}(0, I\sigma)$, where we chose $\sigma = 0.4$ in our experiments. For the initial TS guess, we take $\frac{R+P}{2}$.

V. ADDITIONAL RESULTS

In this section, we present additional results for pre-training on the RQM dataset,[1] and ablations on the proposed GoFlow prior conditioning module and the effect of adding noise to the prior geometry distribution.

RQM pre-training. Results for joint reactant, TS, and product pre-training are shown in Table S2. For TS-only pre-training, results are shown in Table S3. Ground-truth reactant and product geometries were retrieved from the provided IRC dataset by taking the endpoints of the converged IRC calculations and matching them to the reactant or product. For flow matching, the prior distribution was defined by randomly sampling an RDKit conformer and perturbing it with additive Gaussian noise of standard deviation $\sigma = 0.4$. These 32 RDKit conformers per reaction were cached during preprocessing to avoid this computational bottleneck during training.

As the authors of the RQM paper stated, exhaustive transition-state conformer sampling was not performed due to the associated computational cost. Consequently, multiple valid TS conformations may exist for a given elementary reaction step, while only a single structure is provided as ground truth in the dataset. In contrast, smaller datasets such as T1X[2] or RDB7,[3] which involve smaller reacting systems, exhibit substantially less ambiguity in ground-truth TS structures. Consequently, TS prediction errors on the RQM dataset are expected to be higher. The conformational ambiguity is particularly evident in the dihedral errors, which are nearly halved when selecting the generated conformer that most closely matches the ground-truth structure in the dataset. In contrast, the remaining metrics do not exhibit such a pronounced decrease.

TS-only training consistently yielded slightly better results than joint training in our experiments. However, we emphasize that no extensive hyperparameter optimization was performed for the joint training architecture.

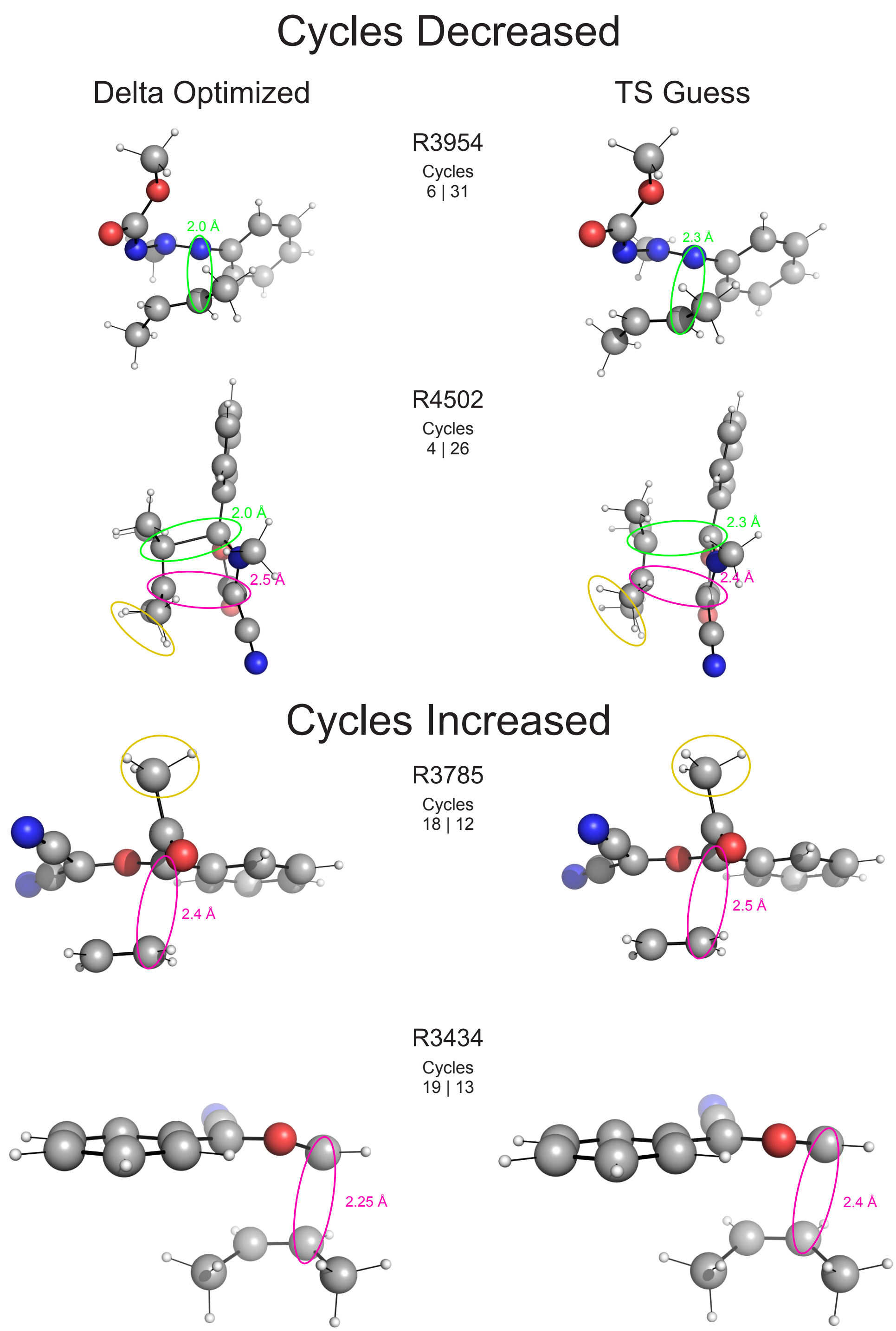

Figure S2. Improved and worsened cases.

Fine-tuning. Ablation results for fine-tuning TsOptNet are provided in Table S4. Adding noise and prior conditioning performed best. See the discussion section in the main paper for more details and interpretation.

Pipeline module by module visualizations. To visualize the flow of information within the pipeline stages we furthermore visualize molecular conformations from each stage of the pipeline in Figure S4. This shows, for an exemplary reaction, which conformations reach the next stage, which are discarded, and the conformational diversity at each stage of the pipeline. Shown are the reactant complexes from the complex finder, TS guesses from the RMSD-pp path search, TS conformers from racerTS, and finally the saddle point optimized TS. Of the shown reactant complexes, two yielded a valid reaction path, while, from their respective low-energy conformers obtained via racerTS, only one yielded an index-1 saddle point. The other TS conformer had two negative imaginary frequencies after saddle point optimization.

E2 reaction conformations. In Figure S3, we show the distribution of the H–C_β–C_α–LG dihedral angle, which determines whether a candidate TS proceeds through a syn- or anti-periplanar elimination pathway. The dihedral angle ranges from $-180°$ to $180°$, and we report its absolute value.

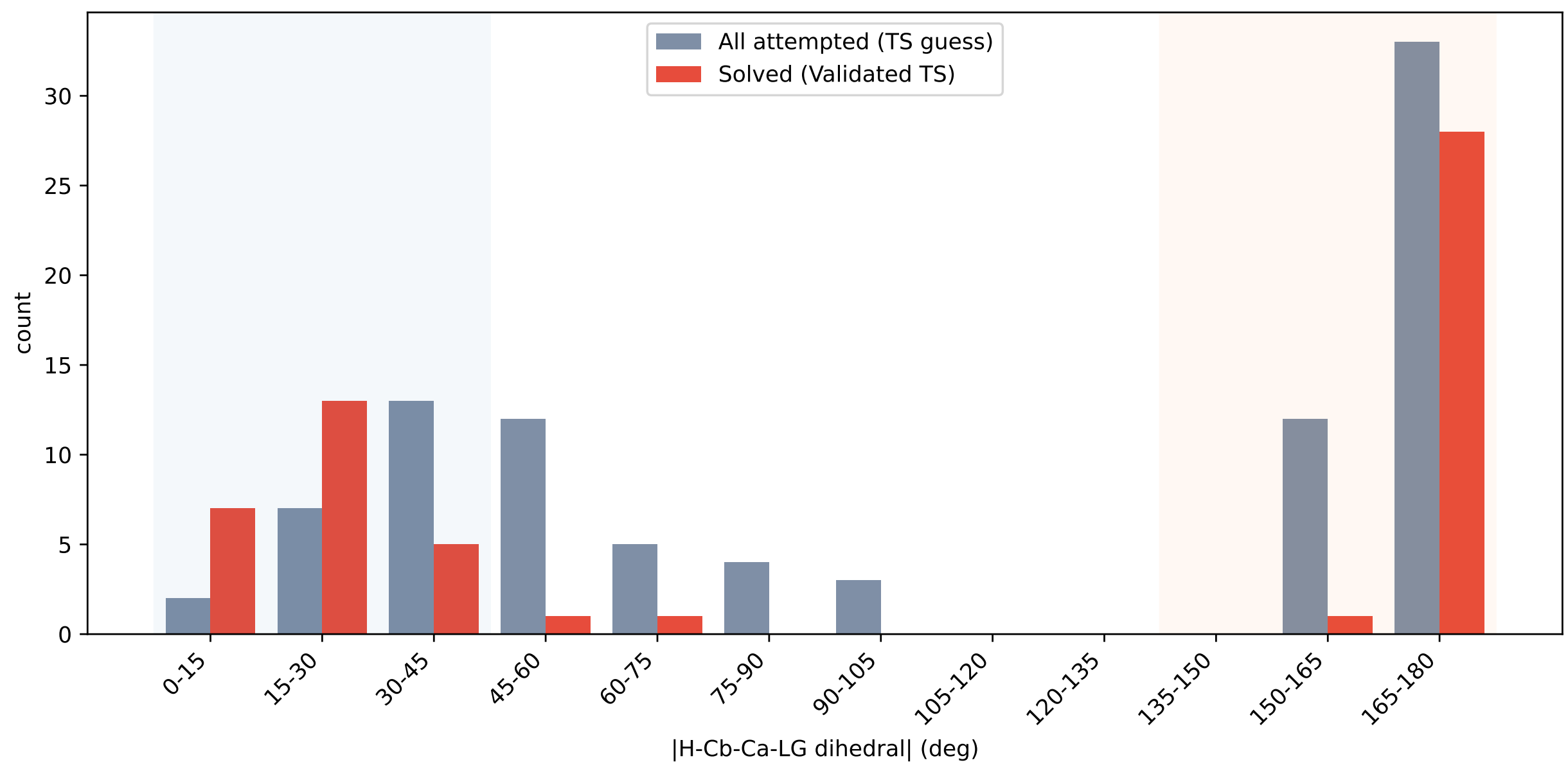

Figure S3. Distribution of the H–C_β–C_α–LG dihedral angle across all attempted $E2$ TS candidates (gray, $n = 137$) and those that converged to an IRC-validated TS (red, $n = 74$). Shaded regions mark the syn-periplanar ($\leq 45°$) and anti-periplanar ($\geq 150°$) sectors. The dihedral angle ranges from $-180°$ to $180°$, and its absolute value is reported.

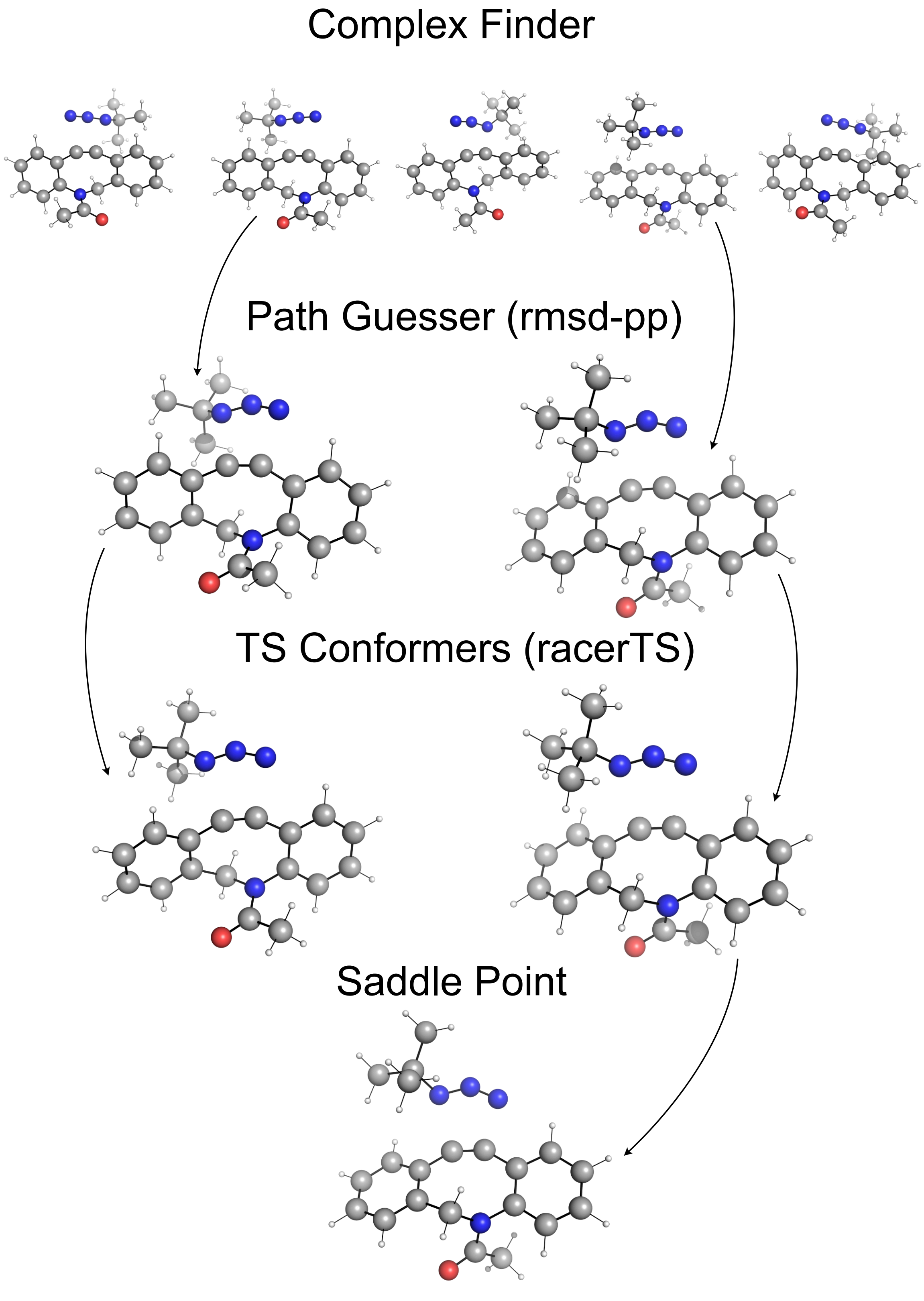

Figure S4. Pipeline module by module visualizations of the ADIBO-tAz reaction.

Head	D-MAE (Å)	RMSE (Å)	Angle (°)	Dihedral (°)
**Reactant (R)**				
Best-of-all	0.261	0.401	3.69	24.51
Median-of-all	0.363	0.599	5.45	44.54
Random-of-all	0.383	0.642	5.57	47.64
**Transition State (TS)**				
Best-of-all	0.234	0.346	4.19	21.32
Median-of-all	0.317	0.521	5.86	37.05
Random-of-all	0.339	0.577	6.10	41.88
**Product (P)**				
Best-of-all	0.262	0.402	3.73	24.79
Median-of-all	0.364	0.599	5.49	44.87
Random-of-all	0.384	0.642	5.62	47.90

Table S2. Performance of the multi-head GoFlow model for Reactant (R), Transition State (TS), and Product (P) geometry prediction on the RQM dataset.[1] We report mean absolute error of interatomic distances (D-MAE), RMSE, angle error, and dihedral error across 15,096 test reactions. For each reaction, we sampled 25 outputs. Results are shown for best-of-all samples, median-of-all aggregation, and random-of-all samples. Best-of-all corresponds to oracle selection across generated samples, and random-of-all to randomly selecting one out of the 25.

Aggregation	D-MAE (Å)	RMSE (Å)	Angle (°)	Dihedral (°)
Best-of-all	0.215	0.322	3.94	19.81
Median-of-all	0.304	0.508	5.45	35.86
Random-of-all	0.332	0.571	5.73	40.77

Table S3. Transition state (TS) geometry prediction performance of GoFlow on the RQM dataset.[1] We report mean absolute error of interatomic distances (D-MAE), RMSE, angle error, and dihedral error across 15,096 test reactions. Results are shown for best-of-all samples (oracle selection), median-of-all samples (typical inference), and random-of-all samples. Best-of-all corresponds to oracle selection across generated samples, and random-of-all to randomly selecting one out of the 25.

Method	Noise (σ)	Prior Cond.	D-MAE (Å)	RMSE (Å)	Angle (°)	Dihedral (°)
PathGuesser	–	–	0.1242	0.1830	1.419	7.56
TsOptNet	0.0	No	0.0845	0.1144	1.685	5.00
	0.1	No	0.0706	0.1038	0.857	3.82
	0.1	Yes	**0.0637**	**0.0976**	**0.661**	**3.26**

Table S4. Transition state (TS) geometry prediction performance comparing different fine-tuning configurations, specifically analyzing the effects of prior noise (σ) and prior conditioning. Results are averaged over 150 test reactions. We report mean absolute error of interatomic distances (D-MAE), RMSE, angle error, and dihedral error. PathGuesser denotes the initial geometry prior to TsOptNet refinement.

Hyperparameter	Value
Learning rate	1×10^{-3}
Learning rate decay	0.1 (patience = 16)
Weight decay	0.01
Gradient clipping	1.0
Number of ODE steps	25
Number of samples (inference)	25
ODE solver	Euler
Active heads	R, TS, P
Prior modes (R/TS/P)	RDKit / Interpolation / RDKit
Noise level (R/TS/P)	0.4 / 0.4 / 0.4
Hidden atom features	256
Hidden output units	64
Shared interaction layers	3
Head-specific layers	2
Radial basis functions	20 (expnorm)
Cutoff radius	5.0 Å
Max spherical harmonics order ($\ell_{\max}$)	2
Attention heads	8
Normalization	LayerNorm

Table S5. Main hyperparameters used for (pre-)training and inference of the multi-head flow matching model.

REFERENCES

[1]K. Lee, S. Kang, B. Kang, W.-J. Son, S. Lee, D. S. Kim, and W. Y. Kim, "A Comprehensive Dataset of Chemical Reactions Covering Second and Third Row Elements with Million-Scale Quantum Calculations," (2025).

[2]M. Schreiner, A. Bhowmik, T. Vegge, J. Busk, and O. Winther, Scientific Data **9**, 779 (2022).

[3]K. Spiekermann, L. Pattanaik, and W. H. Green, Scientific Data **9**, 417 (2022).
